# Embodiment and Emotional Memory in First vs. Second Language

**DOI:** 10.3389/fpsyg.2017.00394

**Published:** 2017-03-23

**Authors:** Jenny C. Baumeister, Francesco Foroni, Markus Conrad, Raffaella I. Rumiati, Piotr Winkielman

**Affiliations:** ^1^International School for Advanced Studies (SISSA)Trieste, Italy; ^2^School of Psychology, Australian Catholic UniversitySydney, NSW, Australia; ^3^Department of Cognitive, Social, and Organizational Psychology Universidad de La LagunaSan Cristobal de La Laguna Spain; ^4^National Agency for the Evaluation of Universities and Research Institutes (ANVUR)Rome, Italy; ^5^Department of Psychology, University of California at San DiegoLa Jolla, CA, USA; ^6^Faculty of Psychology SWPS University of Social Sciences and HumanitiesWarsaw, Poland

**Keywords:** embodiment, emotional memory, first language, second language, EMG, facial motor resonance, skin conductance

## Abstract

Language and emotions are closely linked. However, previous research suggests that this link is stronger in a native language (L1) than in a second language (L2) that had been learned later in life. The present study investigates whether such reduced emotionality in L2 is reflected in changes in emotional memory and embodied responses to L2 in comparison to L1. Late Spanish/English bilinguals performed a memory task involving an encoding and a surprise retrieval phase. Facial motor resonance and skin conductance (SC) responses were recorded during encoding. The results give first indications that the enhanced memory for emotional vs. neutral content (EEM effect) is stronger in L1 and less present in L2. Furthermore, the results give partial support for decreased facial motor resonance and SC responses to emotional words in L2 as compared to L1. These findings suggest that embodied knowledge involved in emotional memory is associated to increased affective encoding and retrieval of L1 compared to L2.

## Introduction

Theories of grounded, or embodied cognition propose that the role of the body goes well beyond that of an instrument by which our actions are realized. Instead, these theories argue that bodily processes contribute to our perception, feelings, thoughts, and behavior (for reviews, see Barsalou, 2008; Winkielman et al., 2015). In the embodied view, knowledge about emotions is grounded in internal systems including the motor, sensory, and autonomic nervous systems. For example, upon the mere viewing of an emotional word or picture, a series of bodily reactions is evoked: Skin conductance (SC; an indicator of physiological arousal) and heart rate (HR) may increase, and we may produce spontaneous facial expressions reflecting a stimulus’ relevant emotional connotation. For example, the zygomaticus major muscle, involved in smiling, has been shown activate in response to positive stimuli, such as pictures and words (Larsen et al., 2003). This involuntary activation of mimetic muscles, sometimes also called facial muscle resonance, can be measured by electromyography recordings (EMG) from probes placed directly above the facial muscle of interest (Cacioppo and Petty, 1981). The range of emotional stimuli to which the mimetic muscles automatically react includes facial expressions (e.g., Dimberg et al., 2000), emotional tone (Quené et al., 2012), as well as emotional words and sentences (e.g., Foroni and Semin, 2009, 2013; Davis et al., 2015; Foroni, 2015; Fino et al., 2016).

However, some recent studies suggest that these embodied activations are less evolved in a second language (L2) as compared to a first language (L1; see Pavlenko, 2012, for a review). For example, Eilola and Havelka (2010) showed that L1 speakers reacted with higher SC to negative and taboo words compared with neutral and positive words while performing an emotional Stroop task (naming color of emotional words). No such pattern was observed for L2 speakers. In line with this, other studies have demonstrated higher SC responses in late bilinguals when they listened to or rated emotional words, phrases, or reprimands in L1 but not in L2 (Harris et al., 2003; Harris, 2004; Caldwell-Harris and Ayçiçeǧi-Dinn, 2009). A recent study extending this line of research indicated that a reduction in embodied responses to L2 might also reflect only partial activation of facial motor resonance (Foroni, 2015).

The idea that facial motor resonance is less developed in an L2 gains importance when considering that the theories of embodied cognition regard facial motor resonance as an important source of embodied knowledge. It is thought to facilitate affective processing and help us to understand the emotional connotation carried by a given stimulus (e.g., Niedenthal, 2007; Winkielman et al., 2008; Wood et al., 2016). For example, blocking participants’ mimic muscles and thereby hindering their spontaneous facial motor resonance has been shown to impair the recognition of emotional words and other people’s facial expressions, and to increase difficulty in processing of emotional sentences (e.g., Oberman et al., 2007; Niedenthal et al., 2009; Havas et al., 2010; Ponari et al., 2012; Davis et al., 2015). In a recent study (Baumeister et al., 2015), we extended this line of research by showing that blocking facial motor resonance during encoding or retrieval not only interferes with initial recognition of emotional words but also impedes their later retrieval. When facial motor resonance had been blocked during the experiment the usually observed memory advantage for emotional words over neutral ones [Emotional Enhancement of Memory (EEM) effect; see Hamann, 2001, for a review] was hampered.

Considering this latter finding, and bearing in mind that an L2 has been found to only partially evoke facial motor resonance (Foroni, 2015), the question arises as to whether such a presumable ‘disembodiment’ of L2 (see also Pavlenko, 2012) might also reflect in diminished affective processing and a weakened EEM effect for emotional L2 words. Several introspective reports, surveys, interviews, and clinical observations suggest that people remain emotionally distant from an L2 if it was not learned during early childhood^1^ (see Caldwell-Harris, 2015, for a recent review). The cognitive and behavioral effects of this emotional distance to an L2 are well documented (e.g., Colbeck and Bowers, 2012; Keysar et al., 2012; Duñabeitia and Costa, 2015). However, there has been a debate as to whether these behavioral differences also extend to the EEM effect.

If facial motor resonance occurring in response to emotion-laden words is decreased in L2 as compared to L1, then this could interfere with an EEM effect in L2. In line with this hypothesis, a study by Anooshian and Hertel (1994) resulted in an EEM effect for L1 but not for L2. Yet, the absence of an EEM effect in L2 is not a consistent phenomenon. Ayçiçeǧi and Harris (2004) found that both L1 and L2 displayed an EEM effect, and the effect was even stronger in L2. In a follow-up to their research, the authors modified the incidental study task to control how deeply participants processed the emotional connotation of the stimuli. This time, the results revealed an EEM effect for L1 but not for L2, provided that the study phase required deep encoding (Ayçiçeǧi-Dinn and Caldwell-Harris, 2009). A possible explanation for these different results may be that encoding of L2 is more effortful than encoding of L1. When L2 and L1 words are mixed during encoding, as was the case in the procedures employed by Ayçiçeǧi and Harris (2004) and Ayçiçeǧi-Dinn and Caldwell-Harris (2009), the elaborative processes and novelty effects associated with L2 may interfere with the processing of words presented in L1. In order to avoid such carry-over effects and distractive task-switching, it could be important to present words in separate blocks for each language. Furthermore, if deep and elaborative processing is encouraged by task instruction for both L2 and L1, then this may lead to a more comparable encoding depth for L2 and L1. These two aspects have been taken into consideration for the experimental set-up of the present study.

The present study aimed to explore the link between embodied processes and memory for emotional content within the frame of L1 and L2 processing. A group of late Spanish/English bilinguals underwent a classical memory task involving encoding and retrieval of both emotionally charged and neutral words in both languages. Facial muscle EMG activity and SC responses were obtained during the encoding phase, in which participants performed a categorization task, which required them to categorize words into “associated to emotion” or “not associated to emotion.” This specific categorization task was meant to induce deep word processing, thought to encourage participants to internally simulate their emotional content (Niedenthal et al., 2009). We predicted that the processing of emotional L2 words would elicit less facial motor resonance and reduced SC responses in comparison to the processing of emotional L1 words. Since embodied simulations have been shown to be modifiable on different levels, including their magnitude, onset, and duration (Simmons et al., 2008), we theorized that the presumable weaker response of facial motor resonance to an L2 could either lead to lower magnitude, to a delay and abbreviation in the mimetic muscle responses, or both. Furthermore, we hypothesized that any reduction of embodied simulations in L2 will impact the initial perception and later memory of emotional words. Regarding the encoding phase, this means that accuracy for categorizing emotional words should be reduced in L2. The presumable link between facial motor resonance and memory for emotional content (Baumeister et al., 2015) raises the expectation that in terms of the memory performance the EEM would be present in L1 but absent or reduced in L2. Finally, we speculated that participants’ levels of facial motor resonance would covary with the retrieval accuracy of emotional word-types in L1. Since processing information in L2 may depend more than L1 on executive control functions (Keysar et al., 2012), our hypothesis in regard to any correlations between motor resonance activation and memory for emotional words in L2 was less definite.

## Materials and Methods

### Participants

Thirty-two young healthy late bilinguals (17 females; mean age: 26.4 ± 5.2) recruited in Southern California and bordering areas of Mexico participated in the experiment. Participants’ first-learned (native) language was either English (13 participants) or Spanish (19 participants), and they spoke their second language (Spanish or English, respectively) at an advanced level. Participants self-assessed (cf. Marian and Neisser, 2000; Pavlenko, 2005) their levels of speaking, reading, and understanding of L2 on a scale from 1 (very poor) to 10 (perfect) as being on average very good [speaking: *M* = 8.5. *SD* = 1.1; reading: *M* = 8.8, *SD* = 1.1; understanding: *M* = 8.9, *SD* = 1.1; ratings ranged from 7 (good) to 10 (perfect)]. Participants reached fluency in their L2 at a mean age of 15 (*SD* = 6.3; age range from 8 to 32 years). Though, most participants had started learning their L2 in a classroom setting, all participants reported to have spent a minimum of 12 months in a country where their L2 was the native language (either because of immigration or because of a student exchange) and to have reached fluency in their L2 during that time. In a classical 1-min letter fluency task, participants produced on average 11 words in L1 (*SD* = 3.1) and nine words in L2 (*SD* = 3.2). A paired-samples *t*-test showed that this difference was significant, *t*(28) = 2.5, *p* = 0.02. Three participants were excluded from all analyses, two because of insufficient fluency in L2 and one due to self-judged onset of fluency in L2 earlier than age 8. Technical malfunctioning led to a loss of data of three further participants, leaving data of 26 participants for the analyses.

### Stimuli

#### Pilot

All final stimuli were selected from a large pool of words composed mainly by word pairs (English–Spanish translations) from an affective database for English and Spanish words (Conrad et al., n.d.), and some emotional words used in a previous research project. A total pool of 345 word pairs was selected for piloting. Words were pre-selected to be associated to the concepts of Happiness or Anger (see also Niedenthal et al., 2009, for the same emotion concepts) or to be neutral according to the previous ratings they had received. To allow comparability, all words were rated again in both languages by 24 native speakers (12 native English speakers rating the English words; 12 native Spanish speakers rating the Spanish words) in an online survey. Not all participants completed all blocks of the pilot, but all words received ratings by at least 10 participants. Ratings were done in regard to the words’ association to the concepts of anger, happiness, and overall emotionality on a scale from 1 (not at all associated to) to 9 (very much associated to). Further ratings were done by the same native speakers in regard to imageability and arousal, again on scales from 1 to 9.

#### Final Word-Selection

In order to be selected as a stimulus, the happy and angry word-pairs were required to be rated across both languages > 3.5 on the respective emotion scale, to be associated to emotional content in general (rating > 3.0), and to not be associated to the other emotions considered (rating < 2.5). Word-pairs were considered as neutral when they were rated in both languages below 3.3 both on the general and the specific emotionality scales. In this way, 320 word-pairs were selected. Happy words were rated higher on the happiness scale (*M* = 6.8, *SD* = 1.10) than angry (*M* = 1.1, *SD* = 0.13) and neutral words (*M* = 1.7, *SD* = 0.52), and angry words were rated higher on the anger scale (*M* = 6.2, *SD* = 1.5) in comparison with happy (*M* = 1.5 *SD* = 0.60) and neutral words (*M* = 1.5, *SD* = 0.24). Furthermore, neutral words were rated lowest on overall emotionality (*M* = 1.6, *SD* = 0.60) in comparison with both happy (*M* = 5.7, *SD* = 1.6) and angry words [*M* = 6.0, *SD* = 1.5; all *F*s(2,639) > 1006, *p*s < 0.001]. There were no differences between the languages for neither the individual emotion ratings nor in terms of arousal and imageability, frequency or word length (all *p*s > 0.25). Furthermore, the individual word categories did no differ in terms of frequency, word length, or imageability. This was true also when analyzed separately for each language [all *F*s(2,319) < 1.72, *p*s > 0.18], nor when averaged across them [all *F*s(2,639) < 2.0, all *p*s > 0.13]. As expected, neutral words were less arousing than happy and angry words [*F*(2,639) = 379, *p* < 0.001]. Detailed information on individual variable ratings per language and word category are given in Supplementary Table 1. All words were divided into eight wordlists (four English wordlists and four Spanish wordlists). Each wordlist was composed of 20 happy words (e.g., Joy, Sweets), 20 angry words (e.g., Murderer, Harassment), and 40 neutral words (e.g., Code, Subject). These wordlists were assembled to form four sets, each including one English and one Spanish wordlist. An English word and its Spanish translation were never assigned to the same set. The four sets (composed of one English and one Spanish wordlist and thus containing 160 words each) were designed to not differ in word frequency (based on the English and Spanish versions of SUBTLEX; Brysbaert and New, 2009; Cuetos et al., 2011), word length, imageability, and arousal within the English [*F*s(3,319) < 2.0, *p*s > 0.12] and Spanish words [*F*s(3,319) < 1.4, *p*s > 0.26]. Furthermore, English and Spanish emotion-laden words (angry and happy) were separately matched across all four sets in terms of their ratings on the respective emotion-scale (anger- and happiness-scale), whereas neutral words were matched for their average overall emotionality ratings [all *F*s(3,156) < 0.34, all *p*s > 0.80]. Finally, it was ensured that within each set, words differed significantly on happiness, anger, and overall emotionality ratings [all *F*s(2,159) > 247, all *p*s < 0.001]. See Supplementary Table 2 for the full list of words.

## Procedure

The study protocol was approved by the Ethics Committee of the University of California, San Diego. The experiment involved an encoding phase and a surprise retrieval phase on two consecutive days. Instructions were presented in the participant’s native language and described the experiment as investigating the effect of SC on word processing in a native and second language. Participants were not informed about the hypothesis related to facial muscle activity and about the subsequent memory test.

### Encoding Phase

Upon arrival for the first session, participants first read and signed a consent form and then completed the Language Experience and Proficiency Questionnaire (LEAP-Q; Marian et al., 2007) to assess their language ability in L1 and L2. Potential baseline differences in daily affect and mood were assessed by the Positive and Negative Affect Schedules (PANAS; Watson et al., 1988), presented in the respective L1 (for the Spanish version, see Sandín et al., 1999). To lower skin impedance for better EMG signals, the skin areas above the zygomaticus major and corrugator supercilii were prepared with cotton pads and abrasive lotion. Residue of the abrasive lotion was removed with cotton pads soaked in alcohol. Bipolar 4-mm EMG electrodes were attached to the corrugator supercilii and the zygomaticus major on the left side of the face. SC was recorded with gold-plated electrodes on the index and middle fingers of the non-dominant hand.

During the first session, participants performed a classification task, requiring them to categorize words into “associated to emotion” or “not associated to emotion” by pressing one of two buttons on a keyboard. Labels to the left and right of the computer monitor served as reminders of the button-assignment throughout the experiment. The English and Spanish word lists of one of the four sets were presented in separate blocks on a computer screen, divided by a short break (5 min). The order of word-lists (English word list vs. Spanish word list) within each set and the set presentation itself were randomized across participants. Each trial started with a fixation-cross presented for 2 s. This was followed by the randomly ordered display of the word stimulus for 3 s and was concluded with the display of a question mark, all presented via E-Prime software (Psychology Software Tools, Pittsburgh, PA, USA). To avoid movement interference with the EMG recordings, participants were instructed to wait for the question mark to give their response. After the response, the next trial started. Every participant started with a short practice session (six trials).

### Retrieval Phase

The second session took place 24 h later and started with participants filling out the PANAS for a second time. This was followed by a surprise memory task. Words from the old and the new set were mixed and randomly presented within a Spanish and English block, providing an equal number of old and new words. Each word was presented individually on a computer screen. Participants’ task was to indicate whether the word had been presented during the encoding phase (old) or whether it was new (new) by pressing one of two buttons. Signs left and right of the computer screen indicated the button-assignment and served as reminders.

### EMG/SC Acquisition

Electromyography and skin conductance signals were recorded using BIOPAC MP150 modules (BIOPAC Systems, Inc., Goleta, CA, USA) set at a sample rate of 2,000 samples per second with gain set to 100. The signals were filtered using a bandpass from 20 to 400 Hz and a notch filter at 60 Hz. The acquisition of the EMG signals was controlled by BIOPAC’s AcqKnowledge software Version 3.0.21 (Mindware Technologies LTD., Gahanna, OH, USA).

## Results

### PANAS

The self-ratings of the PANAS revealed no significant differences between the first and second session of the experiment (*p*s > 0.25), and were thus disregarded in further analyses.

### EMG/SC Data Preparation

Offline processing of the EMG data was performed with software from Mindware Corporation. EMG signals were rectified, integrated, and averaged for a period of 2500 ms in chunks of 500 ms after stimulus onset. Data were cleaned for each participant and each muscle individually, removing data points above or below three SD of participant’s mean. The baseline (recorded during the presentation of the fixation cross, 500 ms before the target) was subtracted from the mean activity. The baseline-corrected score was used as the dependent variable. The same cleaning was applied to the SC data, with the only difference that due to their usual delayed by around 1–3 s after cue onset (see Dawson et al., 2007), SC signals were averaged in chunks of 500 ms for a time period from 1500 to 3000 ms after stimulus onset. For the SC, the root mean square was calculated and all data were standardized. All EMG and SC data associated to trials in which the word presented was incorrectly categorized during the encoding phase were excluded from the analyses (8%).

### Statistical Analysis EMG Data

**FIGURE 1:**
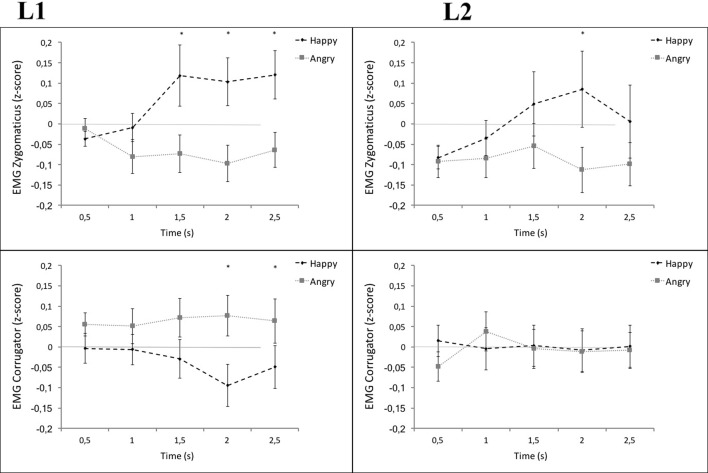
**Mean zygomaticus **(Upper)** and corrugator activity **(Lower)** by time as a function of word-type and language.** Figures show electromyography (EMG) activity in response to happy words (black) vs. angry words (gray) in L1 (left panel) and in L2 (right panel). Error bars represent ±1 SEM. ^∗^*p* ≤ 0.05 (two-tailed).

To examine the relationship between EMG data, language, and word-type, we ran two separate repeated measures ANOVAs one for each muscle (zygomaticus and corrugator; see **Figure 1**). The within-subjects factors were language (L1 vs. L2), word-type (happy vs. angry vs. neutral), and time-point (0–500 ms, 500–1000 ms, 1000–1500 ms, 1500–2000 ms, 2000–2500 ms). The Greenhouse–Geisser epsilon correction was applied throughout all analyses to adjust the degrees of freedom of the *F*-ratios when necessary. Furthermore, all *p*-values of paired-samples *t*-tests are 2-tailed and were Holm–Bonferroni corrected. For the zygomaticus muscle, this analysis revealed a strong effect of word-type [*F*(2,40) = 5.80, *p* < 0.01, $\eta_{p}^{2}$ = 0.19] and a strong interaction of word-type and time-point [*F*(3,67) = 5.32, *p* < 0.001, $\eta_{p}^{2}$ = 0.18]. The interactions between language and word-type [*F*(2,50) = 0.15, $\eta_{p}^{2}$ = 0.006] or between language, word-type, and time-point [*F*(4,97) = 0.94, $\eta_{p}^{2}$ = 0.04] revealed, however, no significant effects (*p* = 0.87 and *p* = 0.49, respectively). For the corrugator muscle, a marginal interaction between language and word-type was present [*F*(2,47) = 2.47, *p* = 0.09, $\eta_{p}^{2}$ = 0.09]. The expected main effect of word-type remained, despite a medium sized effect, insignificant [*F*(2,39) = 1.29, *p* = 0.28, $\eta_{p}^{2}$ = 0.05]. Also the interaction between language, word-type, and time-point remained insignificant [*F*(8,200) = 0.90, *p* = 0.52, $\eta_{p}^{2}$ = 0.04].

Despite the absence of the expected interactions, the visual inspection of the data (see **Figure 1**) suggested that the zygomaticus and the corrugator muscle are differently activated by L1 and L2, particularly in later time windows. We therefore decided to conduct further analyses with the intention to explore facial muscle reactivity in response to emotional words in L1 and L2 at each time point. In a series of pairwise comparisons, EMG activity in response to happy vs. angry words was compared over the five time intervals. Hereby, the activity over zygomaticus and corrugator muscles was analyzed separately.

#### Zygomaticus

Significant differences in response to happy vs. angry words in L1 were recorded in the time window 1500–3000 ms after word onset. In comparison, the zygomaticus activity for happy vs. angry words in L2, differentiated only in the time window 2000–2500 ms after word onset. This result indicates a later onset and shorter duration of specifiable zygomaticus activity in response to happy vs. angry words in L2 compared with L1 (see **Figure 1**). A direct comparison of the averaged differential activity of the zygomaticus in response to happy vs. angry words in L1 vs. L2 did not reach significance [*t*(25) = 1.02, *p* = 0.22, Cohen’s *d* = 0.26].

#### Corrugator

Exploring the specifiable activity of the corrugator in response to angry vs. happy words across time revealed significant results only within L1. Corrugator activity was stronger in response to angry words vs. happy words in L1, starting from 2000 ms after stimulus onset. No significant differences of corrugator activity in response to angry vs. happy words were observed in L2 (see **Figure 1**). A direct comparison of the averaged differential activity of the corrugator in response to angry vs. happy words in L1 vs. L2 gave some indication that the corrugator activity evoked by L2 words was lower than in L1 [*t*(25) = 1.25, *p* = 0.06, Cohen’s *d* = 0.39].

### Statistical Analysis of SC Data

**FIGURE 2:**
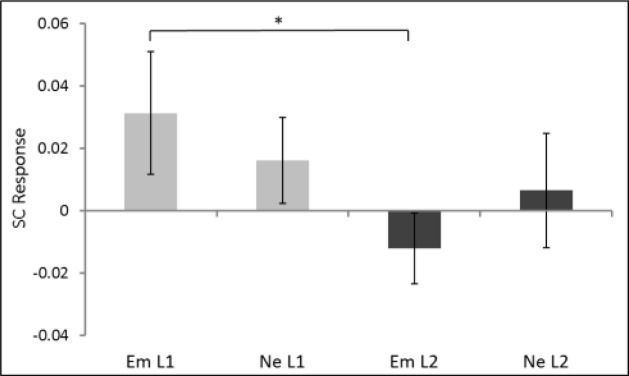
**Skin conductance (SC) in response to emotional (happy and angry together) and neutral words presented in L1 vs. L2 averaged for the period from 1.5 to 3 s after stimulus onset. Error bars represent ±1 SEM.** ^∗^*p* ≤ 0.05 (two-tailed).

Since the Skin Conductance Response (SCR) is only sensitive to arousal but not to different valences, it was averaged for both emotional word-types (happy and angry) within each of the three 500 ms long time windows from 1500 to 3000 ms after cue onset. SCR-scores were submitted to a repeated-measures ANOVA with language (L1 vs. L2), word-type (emotional vs. neutral) and time point (1500–2000 ms, 2000–2500 ms, 2500–3000 ms) as within-subjects factors. The analysis resulted in a marginal three-way interaction [*F*(1,30) = 3.3, *p* = 0.07, $\eta_{p}^{2}$ = 0.12] and a significant interaction between language and emotion [*F*(1,25) = 4.5, *p* = 0.05, $\eta_{p}^{2}$ = 0.15]. We hence continued the analysis by disregarding the factor time point and averaged the SC responses across the time windows from 1500 to 3000 ms post cue onset (**Figure 2**). Pairwise comparisons with these values revealed a significantly stronger SCR to emotional words in L1 (*M* = 0.03, *SD* = 0.10) in comparison with L2 [*M* = -0.01, *SD* = 0.11, *t*(25) = 2.2, *p* = 0.04, Cohen’s *d* = 0.68]. No differences between L1 (*M* = 0.02) and L2 (*M* = 0.01) were present for the neutral words [*t*(25) = 0.43, *p* = 0.67, Cohen’s *d* = 0.12].

### Statistical Analysis of the Behavioral Data

#### Encoding Phase

The dependent variable was the accuracy in discriminating emotional words from neutral words, expressed by the sensitivity index *d’* (Green and Swets, 1966/1974). A paired-samples *t*-test showed that performance was significantly better in L1 than in L2 [*t*(28) = 2.1, *p* = 0.05, Cohen’s *d* = 0.38]. Since it could be argued that this was simply due to lower proficiency rather than reduced emotionality in L2 we investigated whether any differences in response bias, as calculated by the measure *c*, would be present for L1 and L2. The criterion c is defined as the distance measured in SD between the criterion and the neutral point, where neither response is favored. This analysis revealed a subtle bias toward judging words to be neutral in L2 (*c* = 0.04) and to be emotional in L1 (*c* = -0.30). The difference between these two response tendencies was marginally significant [*t*(28) = 2.0, *p* = 0.09]. To further investigated on the suspected reduced emotionality in L2, two paired samples *t*-tests compared the accuracy in percent for emotional words and accuracy for neutral words separately across languages. As expected, this revealed no significant difference between L1 (*M* = 0.85, *SD* = 0.08) and L2 (*M* = 0.82, *SD* = 0.11) for neutral words [*t*(27) = 1.6, *p* = 0.12, Cohen’s *d* = 0.31]; however, unexpectedly, it also revealed no effect for emotional words [L1: *M* = 0.84, *SD* = 0.12; L2: *M* = 0.80, *SD* = 0.16; *t*(27) = 1.3, *p* = 0.20, Cohen’s *d* = 0.28]. In both cases, L1 showed a tendency for higher accuracy compared with L2.

**FIGURE 3:**
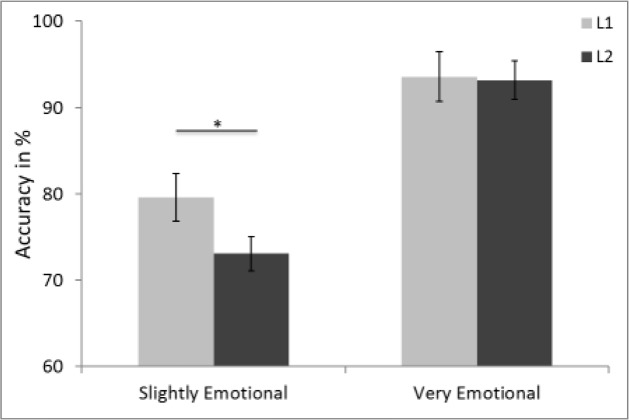
**Accuracy (%) in identifying emotional words as *associated to emotion* during the encoding phase as a function of emotionality (slightly emotional vs. very emotional) and language (L1 vs. L2).** Error bars represent ±1 SEM. *^∗^p* < 0.05 (two-tailed).

Several interpretations of these results seem possible. For example, these results could lead one to assume that the difference in *d*’ might simply be a matter of fluency, which equally affects the neutral and the emotional word-types. However, the marginal response bias toward judging words as emotional in L1 but not in L2 speaks against this explanation. An alternative explanation is based on the fact that the present study used emotional stimuli with different emotional intensity. Some previous studies and reviews have suggested that the facilitative role of facial motor resonance in the encoding of facial expression stimuli may be determined by their emotional intensity (Adolphs, 2002; Oberman et al., 2007; Baumeister et al., 2016). For example, Baumeister et al. (2016) found that blocking facial muscles mainly affected the perception of slightly emotional stimuli. Processing of neutral and strongly emotional stimuli was not at all or less affected. This raises the question of whether a specific impairment in encoding of emotional L2 words was possibly overshadowed by emotional L2 words with stronger emotional intensity. To investigate this possibility, we categorized the emotional words into slightly and highly emotional. Note that the original categorization criteria for the emotional words (described in the subsection, Stimuli) accepted a large scale in emotionality ratings, including words with only moderate emotionality ratings. Thus, to obtain a better understanding of the impact that this broad scale in emotionality ratings for the emotional words might have had, any words with ratings between 3.0 and 6.5 on the overall emotionality scale ranging from 1 to 9 during the pilot were considered to be slightly emotional. Any words with ratings above 7.5 on the same scale were considered highly emotional. See Supplementary Table 3 for the variable ratings of the slightly and highly emotional words and the associated statistical comparisons. To test the prediction that specifically slightly emotional L2 words were impaired during categorization, a 2 (*emotionality*: high vs. low) × 2 (*language*: L1 vs. L2) repeated measures analysis was conducted, with both factors being within-subjects. This revealed a significant effect of *emotionality* [*F*(1,27) = 35, *p* < 0.001, $\eta_{p}^{2}$ = 0.56] and a marginal interaction between *emotionality* and *language* [*F*(1,27) = 3.31, *p* = 0.08, $\eta_{p}^{2}$ = 0.11]. Pairwise comparisons confirmed that participants performed equally well at categorizing highly emotional words in L1 and L2. In contrast, they exhibited a strong bias to incorrectly categorize slightly emotional L2 words as neutral, while this tendency was significantly less pronounced in L1 (see **Figure 3**).

#### Retrieval Phase

**FIGURE 4:**
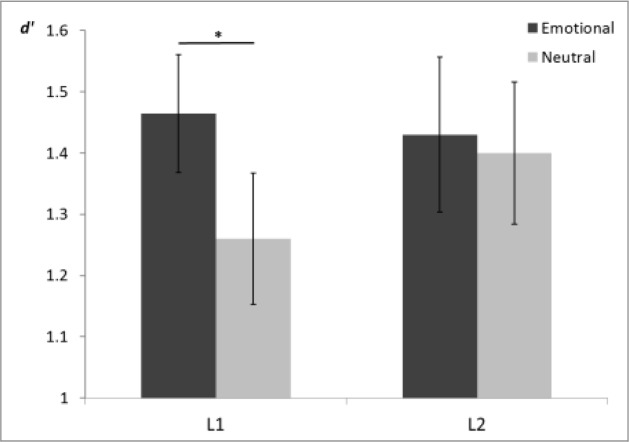
**Memory accuracy expressed in *d’* for neutral and emotional (angry and happy) words.** Error bars represent ±1 SEM. ^∗^*p* < 0.05 (two-tailed).

Since, we were interested in the memory of emotional words vs. neutral words, we controlled for participants’ individual differences in emotion perception by excluding words that had been incorrectly classified during the encoding phase. This was done for each participant individually and guaranteed that our analysis of the memory task for emotional and neutral words was run only on words that had been perceived according to their piloted emotional category (see Baumeister et al., 2015, for a similar procedure). The dependent variable was the memory performance indexed by *d’*, which measures the performance in discriminating between *old* and *new* words. This *d’* index was computed separately for neutral and emotional words, and submitted to a repeated measures ANOVA with the within-subjects factors language (L1 vs. L2) and word-type (emotional vs. neutral). The analysis did not result in any main effects or in an interaction (all *F*s < 2.0, *p*s > 0.18). However, because of the *a priori* interest in the comparison of the EEM effect within L1 and L2, pre-planned pairwise comparisons of emotional vs. neutral words in L1 and L2 were conducted. As expected, participants showed enhanced memory for emotional words (*d’* = 1.46, *SD* = 0.49) in contrast to neutral words (*d’* = 1.26, *SD* = 0.55) in L1 [*t*(25) = 2.20, *p* = 0.04, Cohen’s *d* = 0.38]. Importantly, the EEM effect was absent in L2 [*t*(25) = 0.10, *p* = 0.93], for which participants’ memory for emotional words (*d’* = 1.44, *SD* = 0.65) versus neutral words (*d’* = 1.43, *SD* = 0.59) did not differ (Cohen’s *d* = 0.02). Since the visual inspection of the EEM effects (**Figure 4**) suggested that the reported difference in the EEM effects in L1 and L2 was mainly driven by enhanced memory of neutral words in L2 as compared with L1, a paired sample *t*-test investigated whether this difference was significant. This was, however, not the case [*t*(25) = 1.40, *p* = 0.17, Cohen’s *d* = 0.30]. Finally, two pairwise comparisons showed that the effect size for the EEM in L1 was comparable across angry and happy words, though the *p*-value reached standard significance levels for the happy words only [angry vs. neutral: *t*(25) = 1.80, *p* = 0.08, Cohen’s *d* = 0.44; happy vs. neutral: *t*(25) = 2.50, *p* = 0.02, Cohen’s *d* = 0.46; see **Figure 4**].

#### Correlation between Motor Resonance and Memory for Emotional Words

We further explore the relationship between facial motor resonance and retrieval performance for emotional words by looking at possible correlations between them. For this, we separately averaged the muscle activity recorded between 1000 and 2500 ms after stimulus onset for each muscle (corrugator and zygomaticus) in response to each emotional word-type (happy and angry) and language (L1 and L2) individually for each participant. This time window was chosen since the previous analyses revealed a rather late onset of differential muscle activity (see **Figure 1**). A series of bivariate correlation analyses was conducted to assess whether individual differences in the intensity of corrugator and zygomaticus activation would correlate with the retrieval performance of happy and angry words in L1 and L2. The results did not provide support for any relationship between individual levels of facial motor resonance and the memory for emotional words in L1 nor in L2 [all *r*s(26) < 0.31, all *p*s > 0.13].

Since previous studies suggested that facial motor resonance has a stronger impact on the recognition of slightly emotional stimuli (Adolphs, 2002; Oberman et al., 2007; Baumeister et al., 2016), we were interested in whether similar patterns could be found regarding their memory. For this, we restricted a second series of bivariate correlation analyses to words that had been rated 3.0–6.5 on the general emotionality scale (see section Statistical Analysis of the Behavioral Data). This time the correlation analyses revealed marginal correlations between the levels of zygomaticus activation and the percentage of correctly retrieved slightly happy L1 words [*r*(26) = 0.49, *p* = 0.08], as well as between the levels of corrugator activation and slightly angry L1 words [*r*(26) = 0.35, *p* = 0.09]. Thus, the more zygomaticus or corrugator activation a participant showed during encoding in response to slightly happy and slightly angry L1 words, respectively, the more likely she/he was to remember those words later. A similar non-significant pattern was observed for the levels of zygomaticus activation and retrieval performance of slightly happy L2 words [*r*(26) = 0.42, *p* = 0.10]. No relationship could be found between the corrugator activation and memory for slightly angry L2 words [*r*(26) = 0.01, *p* = 0.97].

## Discussion

The goal of the present study was to test the hypotheses derived from embodied cognition in the context of memory processes for emotional language in L1 and L2. First, the hypothesis that the processing of emotional words in L2 would evoke a lesser degree of embodied simulations compared with L1 was investigated. The results partially supported this hypothesis: Though the expected interactions between muscle resonance and language were not at all or only marginally significant, the visual inspection and pairwise comparisons indicated a tendency for reduced motor resonance in response to emotional L2 words for both corrugator and zygomaticus muscle.

In a second step, the hypothesis that L2 processing would interfere with both the categorization and later retrieval of emotional words was tested. The results were again not decisive but support the notion that L1 but not L2 evokes an EEM effect. Finally, the correlation analyses, gave some indication that participants whose activation of facial motor resonance was strong during encoding were more likely to retrieve emotional words during the memory task. This pattern seemed, however, to be restricted to slightly emotional L1 words and only be partially applicable to L2.

The overall results of the EMG and SC recordings suggested some reductions and differences in embodied simulations of emotional L2 words in comparison with emotional L1 words and extend these results to SC and generalize the results to the corrugator muscle activity not investigated so far in L2 (Foroni, 2015). Even though the expected two-way interaction of *language* and *word-type* was not significant for the analysis of zygomaticus activity and only marginal for the corrugator, some interesting results emerged. For example, whereas pairwise comparisons across time indicated that the activation of the zygomaticus showed typical activity in response to emotional L1 words, these patterns in activation seemed delayed and short-lived in response to emotional L2 words (see **Figure 4**). The difference between L1 and L2 processing became particularly clear in the corrugator muscle, which showed typical response patterns to emotional L1 words but no detectable responses to emotional L2 words. Similarly, the SC was significantly increased in response to emotional words presented in L1 as compared to L2. The direct comparisons of facial muscle responsiveness to L1 vs. L2 revealed a significant difference only for the corrugator muscle but not for the zygomaticus muscles. However, despite not being significant, the zygomaticus showed a similar pattern of decreased responsiveness in L2. Interestingly, Foroni (2015) in testing the somatic correlates of different linguistic forms assessed only zygomaticus muscle activation and reported significant reduction in muscle activation in L2 compared to L1. The present results complement and extend Foroni’s results showing a significant difference for the corrugator muscle and, thus, together they support an embodiment account of emotion processing and a reduced embodiment in L2.

An explanation for the difference between zygomaticus and corrugator muscle activation in L2 found here could lie in the emotional content, which activates the respective muscle. Whereas the zygomaticus reacts to positive stimuli, the corrugator muscle predominantly reacts to negative stimuli. Two recent studies argued that particularly negative words may be at risk of emotional disembodiment during L2 reading, potentially reflecting a positivity bias for L2 processing. A positivity bias is thought to occur if second language acquisition coincides with positive life experiences (Conrad et al., 2011; Sheikh and Titone, 2016). This could be involved in determining the current findings.

Overall, the results of the EMG and SC recordings give some indications that the processing of emotional L2 words is less grounded in embodied simulations than the processing of L1 words. This aligns with previous reports of decreased SC (Harris et al., 2003; Harris, 2004; Caldwell-Harris and Ayçiçeǧi-Dinn, 2009; Eilola and Havelka, 2010) and agrees with studies, which have shown reduced behavioral responsiveness to emotional language in L2 (Colbeck and Bowers, 2012; Keysar et al., 2012). Such results may indicate that L2 learning in adulthood does not necessarily involve the same affective linguistic grounding as L1 learning in childhood. When conceptual and emotion regulation systems have already reached a relatively stable state, the affective grounding of abstract symbols, such as words, may remain shallow. This aspect is particularly relevant for intercultural communication where a weaker somatic base of L2 could on the one hand cause emotional barriers for L2 speakers and on the other hand may be beneficial if emotional distance helps to counteract biases (Keysar et al., 2012; Caldwell-Harris, 2015).

The secondary hypothesis, which states that differences in embodied simulations between L1 and L2 would additionally be associated to performance differences during the encoding and retrieval phase, could be partially supported. During the encoding phase, participants were more accurate at categorizing L1 vs. L2 words. In contrast to the expectation, this effect was caused by an interference with categorizing both emotional and neutral L2 words. This finding opposes the expected difficulty in categorizing specifically emotional words in L2 and could be an indicator of participant’s general lower fluency in their L2. Further investigation and *post hoc* analysis of this alternative account, however, suggested a specific difficulty at identifying slightly but not strongly emotional stimuli in L2, which speaks against a general proficiency effect.

The finding suggesting that the processing of slightly emotional words is particularly linked to motor resonance is in line with the assumption that motor resonance functions primarily as an ancillary information resource, providing additional feedback to the cognitive processes in question (e.g., Adolphs, 2002; Oberman et al., 2007; Oosterwijk et al., 2015). Under clear-cut conditions, the information provided by facial feedback (e.g., Strack et al., 1988; but see also Wagenmakers et al., 2016) may be superseded by dominant established cognitive processes associated to the evaluation of emotional content, reducing its influence on our percept and behavior. However, when the emotional intensity decreases, the interpretation of the emotional stimulus by means of memory and logical reasoning becomes more difficult. Consequently, in order to make a fast and qualified response to a given ambiguous stimulus, we rely more on bodily feedback. In this way, the influence that facial motor resonance has on the interpretation of emotional stimuli increases when cognitive resources reach their limits or leave us uncertain. However, given the *post hoc* nature of this analysis, its results should be interpreted with caution (but see for similar account and supporting evidence Baumeister, 2015). Although stimuli were carefully matched across languages and emotions, the splitting of words in slightly and strongly emotional stimuli was not planned. Therefore, other possible interpretations of the difference we are reporting cannot be completely ruled out. In fact, other variables for which we did not control here may have played a role and future replication or extension of this work should investigate this further. Regarding memory performance, the expected interaction between language and word-type did not appear. Yet, the comparison of memory performance for neutral and emotional words in L1 and L2 supported the initial hypothesis, suggesting the presence of an EEM effect in L1 but not in L2.

Even though the results of the memory task were relatively weak, they align with previous findings reporting no EEM effect in L2 (Anooshian and Hertel, 1994). However, they are in opposition to reports of an equal or even stronger EEM effect for L2 (Harris, 2004; Ayçiçeǧi-Dinn and Caldwell-Harris, 2009). Two reasons could be responsible for these differences in results for emotional memory in L2 reported in the literature. First, it is possible that these differences are owed to dissimilar experimental procedures. For example, while both the present study and the study by Anooshian and Hertel (1994) presented L1 and L2 words in different blocks, the two studies by Ayçiçeǧi and Harris (2004) and Ayçiçeǧi-Dinn and Caldwell-Harris (2009) presented L1 and L2 stimuli intermixed. This may have caused a novelty effect for L2, which may in turn have inhibited normal processing of L1 words. Second, they may be mediated by the distinct circumstances in which participants acquired L2. The Turkish participants in the studies by Ayçiçeǧi and Harris (2004) and Ayçiçeǧi-Dinn and Caldwell-Harris (2009) acquired their second language (English) in the classroom and/or in self-instruction settings and were generally less fluent in L2. Because of this, they may have been more likely to mentally translate L2 words into L1, which may have accounted for the EEM effect in L2. In contrast, most participants in the present study and in the study by Anooshian and Hertel (1994) were exposed to their L2 via immigration or via studies abroad and spoke their L2 with high fluency on a daily basis. It is, however, noteworthy that the current study also encountered large behavioral variances across subjects (see **Figure 4**), which corroborates the assumption that the processing of L2 is determined by more factors besides proficiency.

The reported absence of an EEM effect in L2 is similar to the interference effect observed in our previous study (Baumeister et al., 2015), in which blocked motor resonance specifically inhibited retrieval of emotional words. Those results suggested that facial motor resonance has a crucial role in the processing of emotional content and contributes to the presence of an EEM effect. It is thus of particular interest that the current results gave some indications that facial motor resonance could be reduced when processing L2 words. This suggests that both the external blocking of facial motor resonance and the reduction of facial motor resonance by means of processing in L2 are associated to the absence of an EEM effects.

There are some limitations associated with this study, which should be considered when interpreting the current results. First and foremost, the effects were often subtle and did not always gain the expected significance levels for the interactions. Nevertheless, we decided to continue with pairwise comparisons based on visual inspection of the data. The reason for the weak effect may depend both on the small sample size and the large variations in the data associated to the heterogeneous sample. As mentioned above, the analysis of the slightly vs. very emotional stimuli was explorative in nature and should be interpreted as such. The goal was to direct the reader’s attention to the possibly important aspect of emotional intensity to be considered in future motor resonance studies.

Another subject that awaits further investigation is the determination of the mechanism underlying such reduced affective processing in L2. Lamendella (1977) proposed that implicit linguistic competence, unlike explicit or semantic knowledge, is integrated within the limbic system, involving the striatum and amygdala. This is especially interesting because the amygdala, a structure known to be involved in both experiencing and processing emotional stimuli, shows attenuated activity if facial muscles are blocked by an external force (Hennenlotter et al., 2009). In line with this and the current results, a recent study (Hsu et al., 2014) demonstrated that emotional content in L2 evokes less amygdala activity than it does in L1. Those findings indicate a general interdependence between affective processes, embodied responses, and the recruitment of limbic structures in emotional language processing.

Overall, the results suggest that reading emotional words in a native language provides a deep and embodied emotional experience, which may subsequently also support their salient encoding and retrieval and, as suggested by previous work, even the modulation of subsequent judgments (e.g., Berridge and Winkielman, 2003; Halberstadt et al., 2009; Foroni and Semin, 2011, 2012). The present results together with the results reported by Foroni (2015) suggest that cognitive processes associated with L2 encoding and retrieval seem to be less associated to embodied processes, reinforcing the idea that embodied cognition and emotional memory are linked. Some research already shows how difference in embodiment between L1 and L2 differentially affect individuals (e.g., Puntoni et al., 2009; Keysar et al., 2012) and future research should investigate memory processes as well other domains where the differences between L1 and L2 may have a significant impact implementing other paradigms used to investigate the impact of emotion on behaviors (e.g., Ambron and Foroni, 2015; Ambron et al., 2016).

## Author Contributions

JB designed and ran the study when visiting Winkielman’s lab at UCSD. JB also analysed and wrote up the study. FF, MC, RR, and PW provided feedback on analyses, interpretation, theory, and write-up.

## Conflict of Interest Statement

The authors declare that the research was conducted in the absence of any commercial or financial relationships that could be construed as a potential conflict of interest.

## Footnotes

1 This study only reviews, discusses and draws conclusions from studies in which participants acquired their second language in late childhood, meaning after the age of 8. Whenever we use the term L2 within this study, we refer to a L2 that was learned after the age of 8.
