# Supplementary figures and images for: Embodiment and Emotional Memory in First vs. Second Language
Source: Front Psychol. 2017 Mar 23;8:394. doi: 10.3389/fpsyg.2017.00394 (PMC5362726; doi:10.3389/fpsyg.2017.00394)

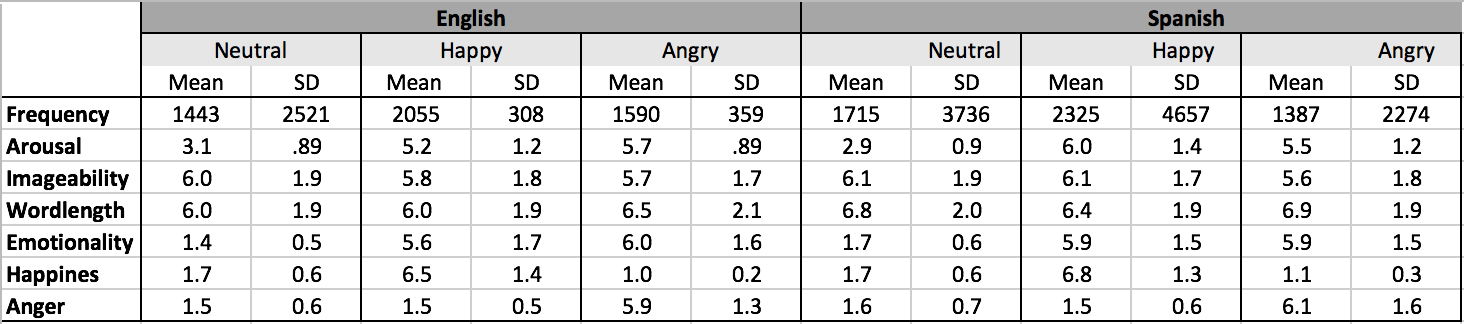


**Table 1:** Variable ratings per language and word category

Supplement: Supplementary file 1 [file Table_1.docx]
