# Supplementary material for: Embodiment and Emotional Memory in First vs. Second Language
Source: Front Psychol. 2017 Mar 23;8:394. doi: 10.3389/fpsyg.2017.00394 (PMC5362726; doi:10.3389/fpsyg.2017.00394)
Supplement: Supplementary file 2 [file Table_2.pdf]

English Words				Spanish Words				Emotion
**Set1**	**Set2**	**Set3**	**Set4**	**Set1**	**Set2**	**Set3**	**Set4**	
Angry	Assault	Broken	Crash	Enfadado	Agresión	Roto	Choque	Angry
Crude	Dagger	Damage	Defeated	Grosero	Daga	Daño	Vencido	Angry
Devil	Displeased	Embarrassed	Failure	Diablo	Desagrado	Penoso	Fracaso	Angry
Fault	Fight	Guilty	Hatred	Culpa	Lucha	Culpable	Odio	Angry
Hell	Hostage	Jail	Killer	Infierno	Rehén	Cárcel	Asesino	Angry
Lie	Loser	Mad	Menace	Mentira	Perdedor	Loco	Amenaza	Angry
Murderer	Nightmare	Obnoxious	Pain	Homicida	Pesadilla	Repugnante	Dolor	Angry
Punishment	Ridicule	Riot	Scorn	Castigo	Burla	Disturbio	Desdén	Angry
Scream	Selfish	Slap	Slaughter	Grito	Egoísta	Bofetada	Matanza	Angry
Slave	Suffocate	Thief	Upset	Esclavo	Asfixiarse	Ladrón	Disgustado	Angry
Useless	War	Waste	Weapon	Inútil	Guerra	Desperdicio	Arma	Angry
Whore	Annoyance	Carelessness	Cheater	Furcia	Fastidio	Descuido	Tramposo	Angry
Cocky	Cuss	Despise	Disingenuous	Engreído	Maldición	Desprecio	Alevoso	Angry
Enraged	Evil	Fist	Gossip	Furioso	Malvado	Puño	Chisme	Angry
Harassment	Jealousy	Lynching	Madman	Acoso	Celos	Linchamiento	Chiflado	Angry
Molest	Nasty	Pollution	Punch	Acosar	Asqueroso	Polución	Puñetazo	Angry
Quarrel	Rampage	Rapist	Scornful	Riña	Enloquecer	Violador	Desdeñoso	Angry
Shotgun	Spanking	Spitefulness	Stalker	Escopeta	Abofetear	Rencor	Acosador	Angry
Stingy	Theft	Threat	Trickster	Tacaño	Robo	Amenaza	Tramposo	Angry
Tyrant	Unfair	Unpitying	Wrath	Tirano	Injusto	Despiadado	Ira	Angry
Aroused	Achievement	Amicable	Amusement	Excitado	Logro	Amistoso	Divertimiento	Happy
Beach	Beautiful	Beauty	Blessed	Playa	Hermoso	Belleza	Bendito	Happy
Bliss	Brave	Breeze	Bride	Éxtasis	Valiente	Brisa	Novia	Happy
Bunny	Butterfly	Caress	Charm	Conejito	Mariposa	Caricia	Encanto	Happy
Cheer	Cheerfulness	Child	Compliment	Ánimo	Alegría	Niño	Cumplido	Happy
Cosy	Cuddle	Cute	Darling	Acogedor	Arrumaco	Lindo	Querido	Happy
Daylight	Dignified	Dream	Elated	Aurora	Digno	Sueño	Eufórico	Happy
Enjoyment	Flirt	Flower	Fun	Goce	Coqueteo	Flor	Diversión	Happy
Gift	Giggle	Greet	Happy	Regalo	Risita	Saludar	Feliz	Happy
Heal	Healthy	Heaven	Holiday	Curar	Sano	Paraíso	Vacaciones	Happy
Hug	Infant	Infatuation	Jewel	Abrazo	Nene	Capricho	Joya	Happy
Joke	Joy	Joyful	Kindness	Chiste	Júbilo	Jubiloso	Amabilidad	Happy
Kiss	Kitten	Loved	Lover	Beso	Gatito	Amado	Amante	Happy
Loyal	Lucky	Masterful	Merry	Leal	Afortunado	Magistral	Alegre	Happy
Mobility	Mom	Ocean	Celebration	Movilidad	Mamá	Mar	Fiesta	Happy
Peace	Pleasure	Puppy	Relax	Paz	Placer	Cachorro	Relajación	Happy
Reward	Save	Savior	Success	Recompensa	Seguro	Salvador	Éxito	Happy
Sunshine	Sweets	Tender	Terrific	Brillo	Dulces	Tierno	Genial	Happy
Travel	Warmth	Wedding	Win	Viajes	Calor	Boda	Ganar	Happy
Wit	Wonder	Yacht	Young	Ingenio	Maravilla	Yate	Joven	Happy
Account	Agency	Alley	Amount	Cuenta	Agencia	Callejón	Cantidad	Neutral
Ankle	Anonymous	Anthem	Arbor	Tobillo	Anónimo	Himno	Glorieta	Neutral
Arm	Aside	Atheism	Avenue	Brazo	Aparte	Ateísmo	Avenida	Neutral

Average	Axle	Banner	Basis	Medio	Axila	Pancarta	Base	Neutral
Basket	Bench	Board	Bourse	Cesta	Banco	Tablero	Bolsa	Neutral
Bowl	Breadth	Broad	Building	Cuenco	Amplitud	Ancho	Edificio	Neutral
Bunch	Cabinet	Candidacy	Chair	Montón	Armario	Candidatura	Silla	Neutral
Chancellor	Chaplain	Chaste	Clock	Canciller	Capellán	Casto	Reloj	Neutral
Cord	Count	Corridor	Cork	Cable	Número	Pasillo	Corcho	Neutral
Curtains	Custom	Decade	Decree	Cortina	Costumbre	Década	Decreto	Neutral
Deity	Disguise	Door	Early	Deidad	Camuflar	Puerta	Temprano	Neutral
Elevator	Engine	Epoch	Evidence	Ascensor	Motor	Época	Prueba	Neutral
Example	Fork	Grail	Gully	Ejemplo	Tenedor	Grial	Barranca	Neutral
Hairdryer	Hairpin	Half	Hat	Secador	Horquilla	Mitad	Sombrero	Neutral
Hay	Hermit	Herring	Icebox	Heno	Ermita	Arenque	Nevera	Neutral
Ink	Item	Jug	Kerchief	Tinta	Pieza	Jarra	Pañuelo	Neutral
Kettle	Key	Law	Layer	Olla	Llave	Ley	Capa	Neutral
Length	Lightbulb	Lighthouse	Location	Longitud	Bombilla	Faro	Lugar	Neutral
Locker	Management	Manner	Market	Consigna	Gestión	Manera	Mercado	Neutral
Month	Opposite	Owner	Paper	Mes	Contrario	Propietario	Papel	Neutral
Part	Passage	Persuade	Pilgrim	Parte	Pasaje	Convencer	Peregrino	Neutral
Plain	Predictable	Prudence	Rank	Llano	Previsible	Precaución	Fila	Neutral
Ratchet	Rate	Register	Replace	Trinquete	Cuota	Fichero	Sustituir	Neutral
Request	Rule	Seat	Solace	Pedido	Regla	Asiento	Consuelo	Neutral
Sort	Square	Statement	Statue	Variedad	Cuadrado	Declaración	Estatua	Neutral
Subdued	Suffice	Syllable	Table	Tenue	Bastar	Sílaba	Mesa	Neutral
Task	Theory	Tool	Truck	Tarea	Teoría	Herramienta	Camión	Neutral
Trunk	Umbrella	Unit	Utensil	Maletero	Paraguas	Unidad	Utensilio	Neutral
Vest	Wagon	Absence	Accumulation	Chaleco	Carro	Ausencia	Colección	Neutral
Address	Ajar	Bland	Blase	Dirección	Entreabierto	Soso	Harto	Neutral
Board	Circumjacent	Container	Corner	Tabla	Circundante	Envase	Esquina	Neutral
Detail	Diver	End	Exchange	Detalle	Buzo	Fin	Intercambio	Neutral
Flat	Foreseeable	Journal	Kettle	Plano	Previsible	Publicación	Tetera	Neutral
Keyboard	Knot	Lawn	Legend	Teclado	Nudo	Césped	Leyenda	Neutral
Loop	Low	Marbled	Measure	Lazo	Bajo	Jaspeado	Medir	Neutral
Middle	Milk	Nun	Oily	Medio	Leche	Monja	Aceitoso	Neutral
Overcast	Size	Screen	Pension	Nublado	Talla	Pantalla	Jubilación	Neutral
Shy	Standard	Skyscraper	Sequence	Vergonzoso	Estándar	Rascacielos	Serie	Neutral
Sphere	Weight	Steep	Slush	Esfera	Peso	Empinado	Aguanieve	Neutral
Tiled	Word	Whistle	Surface	Embaldosado	Palabra	Silbato	Superficie	Neutral

Table 2: List of Words
