# Supplementary material for: Embodiment and Emotional Memory in First vs. Second Language
Source: Front Psychol. 2017 Mar 23;8:394. doi: 10.3389/fpsyg.2017.00394 (PMC5362726; doi:10.3389/fpsyg.2017.00394)
Supplement: Supplementary file 3 [file Table_3.pdf]

	En. words (3.0 - 6.5)						Sp. words (3.0 - 6.5)						En.words (>7.5)				Sp. Words (>7.5)			
	Neutral		Happy		Angry		Neutral		Happy		Angry		Happy		Angry		Happy		Angry	
	Mean	SD	Mean	SD	Mean	SD	Mean	SD	Mean	SD	Mean	SD	Mean	SD	Mean	SD	Mean	SD	Mean	SD
**Frequency**	451	598	1845	2594	1149	1432	2971	4115	1753	3000	1295	2252	1691	2096	1317	1629	1655	2683	4405	7423
**Arousal**	3.9	1.2	5.0	1.0	5.4	0.7	3.4	0.9	5.9	1.5	4.3	1.1	6.4	1.0	6.3	0.7	6.4	1.3	5.2	1.5
**Imageability**	5.8	0.4	5.7	1.9	5.1	1.8	5.3	2.0	5.9	1.7	5.5	1.8	5.8	1.8	4.4	1.3	6.7	1.2	5.1	1.7
**Wordlength**	5.5	0.7	6.1	1.9	6.4	2.2	6.7	0.8	6.5	2.1	6.7	2.0	5.7	2.1	6.8	2.1	6.0	1.7	7.0	2.3
**Emotionality**	3.0	0	4.6	0.9	4.8	1.0	3.1	0.1	5.0	1.0	4.9	1.0	8.2	0.5	8.2	0.4	8.2	0.5	8.0	0.5
**Happines**	2.4	0.5	6.0	1.2	1.1	0.2	1.9	0.6	6.5	1.3	1.2	0.3	7.9	0.8	1.0	0.1	7.9	0.9	1.1	0.2
**Anger**	1.1	0.2	1.6	0.5	5.5	1.2	1.9	0.8	1.6	0.6	5.8	1.6	1.2	0.3	6.4	1.5	1.5	0.5	6.6	1.9

Table 3: Overview of the variable ratings of the slightly emotional words (with ratings between 3.0 - 6.5 on the general emotionality scale) and of the highly emotional words (with ratings > 7.5 on the general emotionality scale) per language and emotion category. Slightly vs. very emotional words did not differ regarding frequency, imageability, and word length neither between nor within languages (all *p*s > .31). The only exception from this was the frequency within the Spanish words, which showed a tendency to be slightly higher for the very emotional words vs. the slightly emotional words (*p* = .09). The ratings on the emotion and arousal scales for the slightly emotional and strongly emotional categories did not differ between languages (all *p*s > .13).
